# What makes health systems resilient? An analytical framework drawing on European learnings from the COVID-19 pandemic based on a multitiered approach

**DOI:** 10.1136/bmjph-2023-000378

**Published:** 2024-03-14

**Authors:** Miriam Reiss, Markus Kraus, Monika Riedel, Thomas Czypionka

**Affiliations:** 1 Research Group Health Economics and Health Policy, Institute for Advanced Studies, Vienna, Austria; 2 Department of Health Policy, London School of Economics and Political Science, London, UK

**Keywords:** COVID-19, Public Health, Epidemics, Communicable Disease Control, SARS-CoV-2

## Abstract

**Introduction:**

The COVID-19 pandemic posed an unprecedented challenge, which caught many health systems widely unprepared. The aim of this research was to develop a comprehensive analytical framework on health system resilience in the context of pandemics. In addition to serving as a tool to analyse the preparedness and resilience of health systems, the framework is intended to provide guidance to decision-makers in health policy.

**Methods:**

The analytical framework was developed based on a multitiered approach. A comprehensive review of the existing literature was conducted to identify relevant frameworks on health system resilience (published between 1 January 2000 and 30 November 2021) and determinants of resilience that emerged during the COVID-19 pandemic. Input was then gathered in several rounds of consultations with designated field experts and stakeholders, drawing on their experiences from the pandemic. Finally, the framework was empirically validated in several case studies.

**Results:**

The framework distinguishes between prerequisites of resilience, pertaining to precautions to be taken in ‘normal’ times, and response strategies in the face of shocks. Both sections are further divided into six building blocks that were adapted from the WHO health system framework: governance and leadership, information and research, financing, physical resources, human resources, and service delivery. An overarching component on contextual factors—subdivided into situational, structural, cultural and international factors—represents an important addition to the existing spectrum of resilience frameworks.

**Conclusions:**

Foundations for a resilient health system must be laid in ‘normal’ times and in all areas of the health system. In the face of a shock, adequate response strategies need to be developed. An essential learning from the COVID-19 pandemic has been that contextual factors of societies and subgroups play a major role in the ability of health systems to overcome a shock, as they impact the implementation and effectiveness of crisis management policies.

**WHAT IS ALREADY KNOWN ON THIS TOPIC**

- Several frameworks on health system resilience have been brought forward in the literature, but the COVID-19 pandemic revealed certain determinants of resilience that had previously not been considered.

**WHAT THIS STUDY ADDS**

- The analytical framework presented in this article builds on previous frameworks and incorporates learnings from the COVID-19 pandemic. In particular, it considers contextual factors of individual societies or subgroups, which have emerged as highly relevant during the pandemic.

**HOW THIS STUDY MIGHT AFFECT RESEARCH, PRACTICE OR POLICY**

- The framework can be used not only by researchers as an analytical instrument when investigating health systems but also by decision-makers in health policy as a guidance tool to assess and improve the resilience of their health system.

## Introduction

 The concept of health system resilience has been the subject of health policy research long before the outbreak of the COVID-19 pandemic, partly induced by previous health crises.^1^,^4^ The COVID-19 pandemic, however, posed an unprecedented challenge that caught many health systems worldwide—even those that had been considered relatively well positioned against health threats^5 6^—widely unprepared.^7^ This novel situation put existing theoretical models of resilience to the test.

Different definitions of health system resilience have been put forward, which the World Health Organization (WHO) summarises as ‘the ability of all actors and functions related to health to collectively mitigate, prepare, respond and recover from disruptive events with public health implications, while maintaining the provision of essential functions and services and using experiences to adapt and transform the system for improvement’.^8^ This raises the question of which features and structures help a health system fulfil these tasks. Pre-existing resilience frameworks provided some valuable insights during the COVID-19 pandemic, but certain aspects only emerged as relevant as the crisis unfolded. We found that, as a result, there was a need for incorporating such aspects in the spectrum of resilience frameworks, as we believe that new perspectives are required to describe and assess what determines the level of resilience of a health system when confronted with a pandemic. In particular, this pertains to what we refer to as contextual factors of societies and subgroups, which have been shown during the recent pandemic to crucially impact the effectiveness of resilience strategies.

The aim of our research was to address this need and contribute to the ongoing discourse by developing a comprehensive analytical framework on health system resilience in the context of combatting a pandemic, drawing on both existing theoretical models and experience from the COVID-19 pandemic. The framework may also to a certain extent be applicable to other health emergencies (eg, climate-related events, nuclear incidents), but its focus lies on infectious disease outbreaks. Notwithstanding, we found that specifically including deliberations on further health emergencies may overstretch the framework and compromise its usability. Furthermore, the framework is predominantly geared toward the European context, given the commonalities between European health systems in terms of, for example, resource availability and coverage. Moreover, an important feature of our framework is the role of contextual factors, which may vary to a substantial extent when taking a global perspective.

In defining what constitutes high performance of a health system, we rely on the goals of health systems that have been established by WHO over decades.^9^ While different versions of these goals have been brought forward over time, WHO has recently summarised them in their health system performance assessment framework for universal health coverage as health improvement, people centredness and financial protection, complemented by the two cross-cutting goals of health system equity and health system efficiency.^10^

In addition to serving as a tool to analyse preparedness and resilience of health systems, the framework is intended to provide guidance to decision-makers in health policy by helping them assess the situation of their health system and prioritise areas of action, both during an acute crisis and in ‘normal’ times. The framework adopts a wide view of the health system and specifically addresses societal context factors, which proved to be of high significance for the effectiveness of pandemic management.

## Materials and methods

### Literature review and first draft

To identify existing frameworks on health system resilience, we searched PubMed and Google Scholar for articles in English or German language published between 1 January 2000 and 30 November 2021. We used the following search string in a title and abstract search: (framework* OR concept* OR model*) AND (health system*) AND (resilien* OR shock* OR preparedness OR cris*). We reviewed the articles to identify those that contained an original framework or type of theoretical model on health system resilience. Articles were excluded if the framework was not applicable to infectious diseases, or if they predominantly pertained to the context of low-income and middle-income countries. In addition to the database search, we manually screened the reference lists of relevant publications and conducted a targeted search of repositories of relevant institutions, namely the European Commission, the European Centre for Disease Prevention and Control (ECDC), the European Observatory on Health Systems and Policies, the Centers for Disease Control and Prevention, the United Kingdom National Health Service and the WHO. In total, we included ten frameworks in our analysis.^12 11^,^18^ A summary table of the main characteristics of the resilience frameworks identified in the literature review can be found in the online supplemental materials.

We analysed the identified frameworks with respect to their underlying concepts, context, target audience and region, phases covered, elements, structure, outcomes and knowledge base. We then assessed their comprehensiveness and applicability in the context of the recent pandemic. We did this by comparing the frameworks and their elements along the lines of two questions: (1) which features of the framework appear particularly useful for analytical purposes and should thus be adopted in our framework and (2) which features could be added or adapted in the framework to better serve our purpose? Based on this analysis of existing frameworks, we devised a fundamental structure for our own framework.

Several frameworks introduced a temporal dimension by defining phases, as, for example, a precrisis, pericrisis and postcrisis phase.^1 12^ The postcrisis phase generally involves recovery and learning from a crisis to improve preparedness for potential future shocks. To emphasise the need for a structural foundation of preparedness, we decided to adapt the temporal dimension towards a distinction of prerequisites of resilience and response strategies in the face of shocks as first level of classification.

A further level of classification is by health-system building blocks. In the World Health Report 2000,^9^ WHO identified basic functions of health systems and later derived from these six essential building blocks: leadership and governance; information; financing; medical products, vaccines and technologies; health workforce; and service delivery.^19^ This set and variations thereof have been widely used in health-system analysis and most of the resilience frameworks we identified address at least some of the building blocks. We drew on the six original building blocks but introduced minor adaptations to better fit the context of pandemics. We broadened the information component to include research, as the latter played an essential role in combatting the COVID-19 pandemic. We extended the medical products, vaccines and technologies component to physical resources in general, as the crisis underlined that managing a pandemic requires a broad range of resources. Finally, as counterpart to physical resources, we used human resources rather than health workforce to again reflect the variety of professions involved in crisis management. The three other building blocks (governance and leadership; financing; service delivery) remained unchanged.

The frameworks included in our review were mostly developed before the COVID-19 pandemic. To identify additional resilience factors that emerged as relevant during the pandemic and, if necessary, adapt those already identified, we conducted a complementary literature search structured along the components of our framework. An individual search was conducted for each component which aimed at detecting scientific backup for (or against) inclusion into the framework, based on empirical findings from the COVID-19 pandemic. This search also revealed that contextual characteristics of individual societies or subgroups played a major role in the ability of health systems to manage the COVID-19 pandemic. We, therefore, introduced contextual factors as a prominent component into our framework. We derived the conceptualisation of contextual factors from the health policy framework by Buse *et al*.^20^

### Internal and external expert consultations

After a draft version of the analytical framework had been created, we continuously developed it further in an iterative process. We held multiple rounds of internal brainstorm sessions to define the individual elements within the sections of the framework, incorporating both elements from the existing frameworks and resilience factors identified in the complementary literature search. The individual elements were defined in accordance with the goals of health systems as stated in the WHO health system framework, namely responsiveness, good health and fairness of financing.^9^

To complement the findings from the literature, we subsequently presented the framework to designated field experts and relevant stakeholders to learn from their experiences during the COVID-19 pandemic. In these consultations, we explained the background of our research to experts, gave a detailed overview of the current version of the framework and asked them for feedback and further aspects they felt were missing. The expert consultations served the additional aim to ensure that the framework was well understood and purposive. After each consultation, the feedback was incorporated into the framework. Experts and stakeholders from the following institutions were consulted: ECDC, European Observatory on Health Systems and Policies, reference network for European Regional and Local Health Authorities, European Hospital and Healthcare Federation and the Austrian COVID-19 Future Operations Platform.

### Empirical case studies

Finally, we empirically applied the framework in three sets of case studies. These were part of a more comprehensive research project^21^ and performed in several European countries to gain insights on experiences during the pandemic. The three sets of case studies investigated primary care systems (Austria, Denmark, France, Hungary, Italy),^22^ hospital care (Denmark, France, Germany, Hungary, Italy)^23^ and public health (Austria, Great Britain, Spain),^23^ respectively. The case studies were based on a total of 107 semistructured interviews with professionals from the respective fields. Data collection and analysis in the case studies was guided by the analytical framework, with the interview guides being designed along the basic structure of the framework. The results of the case studies were, in turn, used to empirically validate and finalise the contents of the framework. The elements included in the framework showed high empirical relevance in the case studies, and the framework proved to be a purposive analytical tool in the research process.

### Patient and public involvement

Patients and the public were not involved in any way in the research process.

## Results

**Figure 1:**
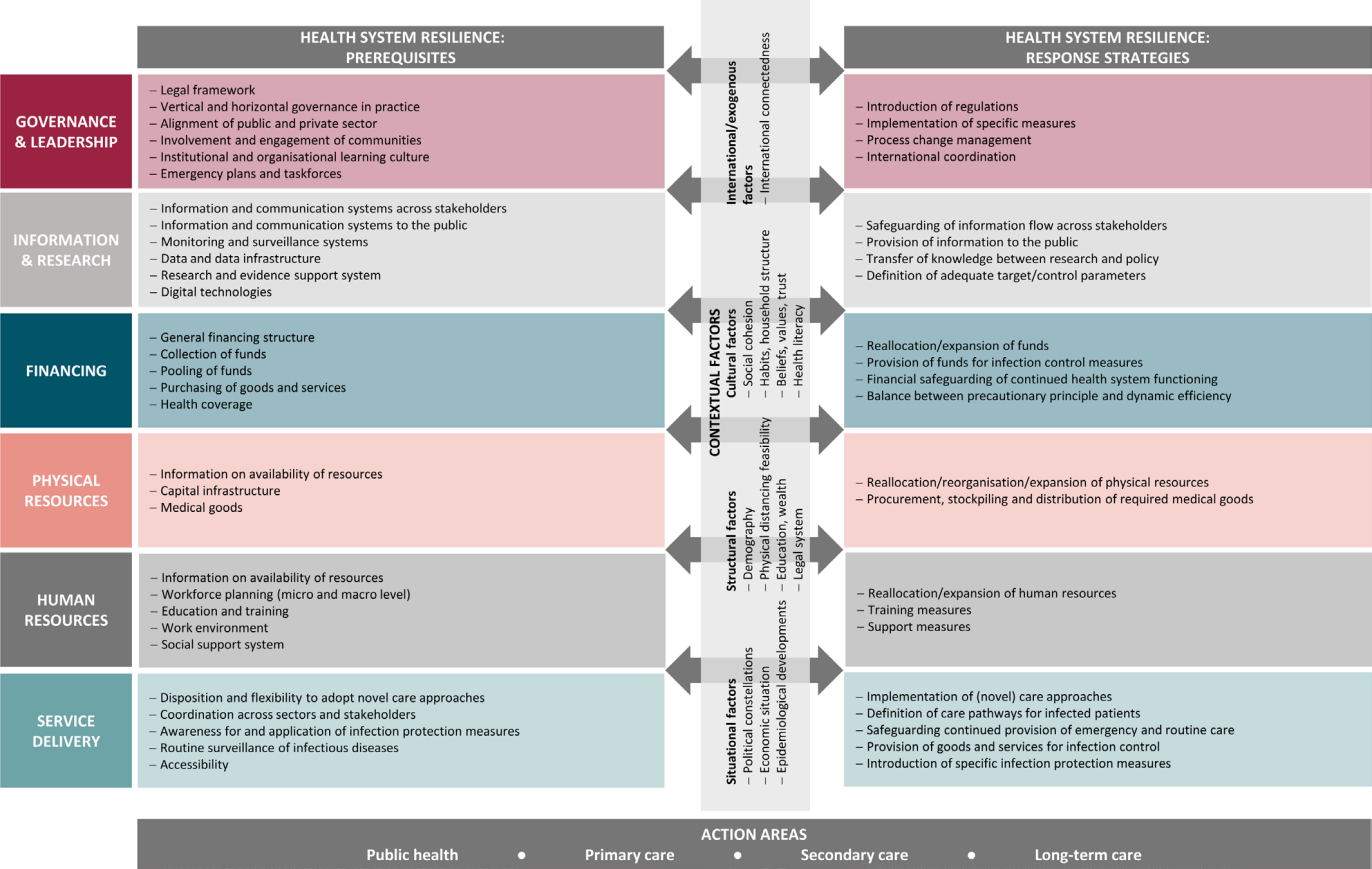
Analytical framework on health system resilience in the context of pandemics.

The analytical framework is presented in figure 1 and is structured along two main dimensions. First, it features a structural dimension distinguishing between prerequisites and response strategies.^1 12^ The former addresses precautions that should be taken in ‘normal’ times and focusses on capacities and structures, while the latter is geared towards active intervention in the event of a crisis. We believe that this distinction is more expedient for analysing resilience than a temporal structure, as the prerequisites usually take longer to change and should therefore be worked on both before and after an acute crisis. Response strategies, in contrast, build on these prerequisites and can be deployed in the short term when the need arises.

Second, it is structured along six building blocks adapted from the WHO health system framework, which should be particularly useful when applying the framework as an analytical tool for assessing resilience in different domains of the health system.^9^ The framework furthermore features a component on contextual factors that act as filters affecting the effectiveness and applicability of resilience strategies. We regard the inclusion of such factors as an important innovation and distinguishing feature in the spectrum of existing resilience frameworks. These factors may help explain why a policy that proved effective in one country or region may not be as effective or not even feasible to implement in a different one—or why the approaches proposed in resilience frameworks may not be applicable to the same extent in every country.

It should be noted that the degree to which the different determinants of resilience are amenable to change through policy measures varies, both by determinant and by the context of the health system. Contextual factors in particular, and, to a lesser extent, prerequisites tend to take time and a certain level of effort to change.

The COVID-19 pandemic demonstrated that health shocks can impact all spheres of the health system, including those that are not typically represented in narrower views of health systems. To account for this, we apply a comprehensive understanding of health systems. Our framework is thus intended to address resilience in several action areas including public health, primary care, secondary care and long-term care.

As mentioned above, we use the goals of health systems as defined by WHO in their health system performance assessment framework (health improvement, people centredness, financial protection, equity, efficiency) as a compass in the development of the framework. The elements within the building blocks were chosen to ultimately serve these goals, even when it is not explicitly stated below.

### Governance and leadership

Health governance systems vary greatly across European countries, each with their own strengths and weaknesses. The challenge of responding to a novel threat such as the COVID-19 pandemic put different systems to the test.^24^

Many health systems in Europe are characterised by a high degree of fragmentation, which has negatively impacted their ability to combat the pandemic.^25^ A sound legal framework—including a clear allocation of competences across stakeholders (eg, levels of government, payers, providers) and a legal basis for crisis management (eg, epidemic laws)—as well as a high level of coordination in both horizontal and vertical governance put health systems in a position to respond more effectively to emerging threats.^26 27^ A specific aspect of this is whether to organise processes in a centralised or decentralised fashion, and who to involve in decision-making. It has become evident during the pandemic that there is no unique recipe in this regard, but that different policy areas may require different approaches.^28 29^ Similarly, there should be good alignment between the public and the private sector in various fields to ensure that an engagement of private sector resources can be organised smoothly in case of a crisis.^30^

A participatory and responsive style of leadership that allows for involvement and engagement of communities increases acceptance of decisions and helps with implementation of policies.^31^ An institutional and organisational learning culture facilitates rapid process change management when required.^32^ Emergency plans and taskforces that are up to date and ready for action enable a swift response.

However, a well-designed governance system is of limited use when it is circumvented in the event of a crisis. Experience during the COVID-19 pandemic has shown that in several countries, governments used the emergency situation to shift power relations and deviated from scientific advice for political reasons.^24 33 34^ When introducing crisis regulations and measures, it is thus essential that decision-makers act transparently and are held accountable for their actions. The regulations and measures themselves (eg, test–trace–isolate–support, physical distancing) should be appropriate to the situation, that is, neither excessive nor insufficient. Finally, since infectious diseases are not contained by borders, the COVID-19 pandemic highlighted that resilient health systems require international coordination and cooperation. Regulations and measures should be internationally aligned to be fully effective. Accordingly, there have been increasing efforts to promote health governance not only on the pan-European but also on the global level.^35 36^

### Information and research

Crises being exceptional situations require intensive efforts not only to retrieve decision-relevant information but also to communicate to all groups in society. The COVID-19 pandemic is said to have been accompanied by a so-called infodemic, as information is increasingly available in real time and on a variety of platforms.^37^ Despite, but also owing to, this broad availability of information, it has been a major challenge in pandemic management to get the right information across to the right recipient.^38^ Effective information and communication systems—both across stakeholders and to the public—should already be established in normal times and then further extended when needed to allow for a timely flow of information. This includes risk communication towards the public, which has been shown to significantly affect preventive behaviour during the COVID-19 pandemic.^39^ Risk communication should be transparent, clear and easily accessible and understandable for laypersons.^40^ Evidence from the COVID-19 pandemic has made clear that information campaigns should be tailored to the needs and capabilities of different groups, taking into consideration aspects such as health literacy levels or prevailing beliefs.^41^ This is of particular importance when aiming for equity in information provision, since groups that are more vulnerable to begin with tend to be harder to reach with such campaigns.^42^

Another crucial aspect is provision of relevant and reliable information to healthcare providers.^43^ Since the state of knowledge often changes quickly during a crisis, this uncertainty should be taken into account and communicated clearly and transparently (best available evidence).

Monitoring and surveillance systems should be in place that are practicable and adaptable to new risks. This requires a comprehensive and up-to-date data infrastructure ensuring that threats are identified in time and critical decisions made accordingly. In the same way that governance should be coordinated internationally, monitoring and surveillance systems should be linked and data exchanged across countries.^44^ A major challenge during the COVID-19 pandemic has been that epidemiological data—if available—were often not standardised, which made it difficult to combine or compare data from multiple sources, both within and across countries.^45^

Data availability is also an essential prerequisite for research in various fields (eg, epidemiology, virology, public health, operations management). The basis for this—in the form of research infrastructure and supportive conditions—needs to be built in normal times. This also involves the build-up of a comprehensive evidence support system that facilitates mobilisation of knowledge generated by research—both to policy-makers and to the public.^46^ Certain levels of health literacy and trust in science are required for this to be effective, which is further discussed in the section on contextual factors. In case of a crisis and building on these preconditions, the constantly evolving evidence should be transferred to and thoroughly reviewed by policy-makers to enable evidence-based decisions and definition of adequate target and control parameters. Such parameters (eg, level/development of daily new cases, bed capacities, vaccination rates) can help monitor relevant changes and should be uniformly used by all stakeholders.^47 48^

Digital technologies play an increasingly crucial role for health system resilience. Various forms of technologies (eg, data transfer systems, smartphone apps, telehealth solutions) have been (further) developed and used during the COVID-19 pandemic for a wide range of purposes (eg, contact tracing, information sharing, provision of healthcare, infection surveillance).^49^,^51^ There is little doubt that digital technologies have the potential to be an important asset for health systems during normal times and crises. However, some applications have raised concerns about data privacy and security and thus need to be embedded in a sound legal framework.^49 52^

### Financing

Health systems generally benefit from a clear, sustainable and flexible financing structure. In the face of a shock, this becomes even more relevant as funds need to be reallocated and expanded adequately. This involves additional resources for infection control measures such as testing, personal protective equipment and vaccinations, as well as safeguarding of sufficient financing for continued health system functioning (eg, remuneration schemes). Uncertainty with respect to financing may hamper and slow down response measures.

Health financing consists of three subfunctions: collection of funds, pooling of funds and purchasing of goods and services.^53^ Collection of funds in a resilient health system should be stressable, ideally drawing on a broad and crisis-proof funding source and equitable.^54^ As public revenue used for health financing is mostly cyclical, many countries were forced to broaden their financial base during the COVID-19 pandemic and previous crises to generate sufficient funds. This holds true in particular for countries with social health insurance schemes, where insurance contributions are strongly dependent on the labour market situation.^55^ In federal countries where subnational governments play a major role in health service provision, the alignment of centralised and decentralised funding also has to be taken into account.^56^

Pooling of funds should be inclusive with respect to risks and income levels. Purchasing of goods and services should be equitable while ensuring allocative efficiency and dynamic cost-efficiency. The COVID-19 pandemic critically impacted purchasing patterns and in many countries, purchasing decisions were partly centralised to federal governments, especially regarding public health services.^57^ Many decisions involved a trade-off between the so-called precautionary principle and dynamic efficiency. The former, in this context, refers to the application of restrictive and/or costly measures (eg, reserve capacities in hospitals, broad testing campaigns) when conclusive evidence on their effectiveness is still lacking.^58^

While universal health coverage may be controversial in some countries and for some types of services, health services related to infectious diseases should be broadly covered to ensure low-threshold access.^59^ This particularly applies to services aimed at infection control such as vaccinations and testing, as they exhibit positive externalities. Many countries extended entitlement to services related to COVID-19 and/or exempted them from user charges.^55 60^

### Physical resources

Health system capacities were strained to an unprecedented extent during the COVID-19 pandemic. For decision-makers to be able to plan effectively and make adjustments when needed, it is first of all necessary to have up-to-date information on the availability of resources. This applies both to capital infrastructure (eg, hospital capacities, digital technologies, production facilities) and medical goods (eg, personal protective equipment, medication). This emerged as a problem during the pandemic when in several countries, data on hospital and intensive care unit (ICU) capacities were either of insufficient quality or lacking altogether.^61^

A certain level of physical resources required for managing crises needs to be accumulated in normal times while maintaining that these resources consistently meet quality and safety standards. The exact level of resources to be held is, however, subject to controversy. While evidence suggests that higher hospital (in particular, ICU) capacities tend to have been associated with lower total COVID-19 mortality,^61 62^ it remains unclear what level of reserve capacities is optimal. With regard to medical products, researchers and policy-makers have been developing strategies to stockpile essential medical goods and make supply chains more crisis resistant.^63^,^65^

In the face of a shock, existing physical resources must be reallocated and reorganised in a timely and efficient manner and, if required, additional resources must be acquired. During the COVID-19 pandemic, hospital departments were repurposed or closed, and elective surgeries postponed to free up capacities. To minimise disruptions to service delivery, reorganisation efforts should be well coordinated.^13 66 67^ Medical goods required to manage the crisis (eg, personal protective equipment, vaccines) have to be procured, stockpiled and distributed according to need. Furthermore, the provision of resources should be well aligned between the public and the private sector. Many European health systems had to increasingly engage private sector resources to ensure that needs were met during the crisis, for example, by procuring medical goods from private sector suppliers or making use of capacities of private providers.^30^

### Human resources

The COVID-19 pandemic has demonstrated that even ample physical resources are not sufficient to manage a crisis when there is a lack of human resources.^68^ This became most visible in hospital care, but applies to a similar extent to public health, primary care and long-term care. In addition, it has to be safeguarded that the workforce is not only sufficient in quantitative terms but also receives adequate training and faces supportive working conditions.

Information on the availability of human resources is vital for planning both on the macrolevel, that is, entire health systems or sectors, and on the microlevel, that is, individual organisations. Planning should be sustainable and foresighted while allowing for enough flexibility to adapt to situations of changed demand.^69 70^ This involves optimal provision of education and training, which should encompass crisis management and personal resilience skills.^69^

In the event of a shock, workforce has to be reallocated, reskilled and potentially expanded, which was widely done during the COVID-19 pandemic.^67 71^ Considering time pressure, such measures should be implemented in an efficient and well-planned manner—but always under consideration of health workers’ well-being. Working in the health sector (including informal caregiving^72^) is generally physically and mentally demanding, but there is ample evidence suggesting that the pandemic multiplied pressures in many areas and caused substantial psychological distress among health workers.^73 74^ Workers should thus receive additional support of various kinds during a crisis, including not only mental health support but also financial compensation for their increased workload.^67 75 76^ Human resources in the health system in a wider sense also include a social support system consisting of, for example, informal caregivers and volunteers. These should be well embedded in the larger system and included into resilience strategies.

### Service delivery

The COVID-19 pandemic presented health systems with the dual challenge to provide adequate care to at times overwhelming numbers of COVID-19 patients while upholding service delivery for non-COVID-19 patients. To be able to rise to such a challenge, health systems should fulfil certain prerequisites. The system in general and providers in particular should have a certain disposition to adapt to rapidly changing circumstances and adopt novel care approaches when necessary.^77 78^ This pertains, for example, to the use of e-health tools,^51^ but also to services required for infection control (eg, testing, vaccinations).

Sectors and stakeholders should be well coordinated already in normal times to enable a smooth adaptation of the care process in case of a shock. During the COVID-19 pandemic, new pathways had to be defined for infected patients including testing, symptom monitoring and treatment. This required public health authorities, primary care providers and hospitals to align their services, which turned out to be challenging in many countries.^79^ Cross-sectoral collaboration is not only important within the health system, but service delivery should also be aligned with sectors and policy areas beyond the health system, such as education, social services and employment. This has proved particularly important to mitigate the adverse impacts of the COVID-19 pandemic, especially on more vulnerable groups.^80 81^ Furthermore, service delivery should be well coordinated between the public and private sector, since a crisis may require that the additional or changed demand for services in various areas is partly covered by private sector providers, as has been the case during the COVID-19 pandemic.^30^

While managing service delivery for infected patients in a pandemic, health systems also have to ensure continued provision of emergency and routine care. During the COVID-19 pandemic, most countries were forced to prioritise essential services and postpone non-urgent care such as preventive screenings or elective surgeries. Some services were transferred from inpatient to outpatient settings or provided remotely.^60 82^

Safety in service delivery should always be a priority. Healthcare providers should have a high awareness for infection protection and apply certain preventive measures also in normal times. During a crisis, safety in service delivery should be ensured by introduction of adequate protective measures (eg, testing requirements, masks, separate consultation hours for potentially infectious patients) and provision of sufficient protective equipment.^79^ To detect emerging threats in a timely manner, routine infectious disease surveillance should be in place.^83^

Generally, health services should be broadly accessible at all times, especially for vulnerable patient groups. This is even more important during a health crisis. For this reason, many countries granted low-threshold access to services related to infection control and treatment of COVID-19 during the recent pandemic.^60^

### Contextual factors

In our analysis of existing resilience frameworks, we found that most frameworks appear to be designed as universal templates to be applied to any health system. The COVID-19 pandemic, however, has unveiled that contextual factors of individual societies or subgroups play an essential role in the implementation and effectiveness of pandemic management policies. They can act directly as determinants of health system resilience or indirectly as filters through which prerequisites for resilience and response strategies interact.

Buse *et al*^20^ discuss four types of contextual factors that may impact health policy, which were originally introduced by Leichter^84^: situational factors, structural factors, cultural factors and international/exogenous factors.

Situational factors are transient or idiosyncratic conditions leading to policy changes, including specific political constellations^85^ or short-run economic fluctuations. The pandemic itself, particularly the timing and intensity of its waves, which varied significantly across countries, can also be regarded a situational factor resulting in different policy responses.

Structural factors are more permanent features of a society and may pertain to, for example, its political system, economy, state of development or demography. The latter, in particular age structure, has played an important role during the COVID-19 pandemic, as older age is a major risk factor for severe disease and younger age has been associated with increased spread, especially in schools.^86^ The feasibility of physical distancing, determined by, for example, population density or dependence on public transport, has been a notable structural factor.^87^ Another example is the education system, as education affects health literacy and comprehension of risks. More wealthy countries, furthermore, tend to be less vulnerable to shocks,^88^ as a favourable economic position may facilitate both the build-up of prerequisites and the adoption of (costly) response measures. A country’s legal system is also a crucial structural factor for pandemic management: certain policy measures taken in the COVID-19 pandemic conflicted with fundamental rights such as data protection and freedom of movement—in some countries more than in others, depending on their legal systems.^89 90^

Cultural factors are more difficult to grasp and can take on many forms. Social cohesion within a society has been suggested to facilitate recovery from a crisis.^91 92^ Another example of a relevant cultural factor in the context of infectious diseases are social habits regarding physical contact such as greeting customs.^93^ Household structure can also play an important role in this context: patterns of cohabitation vary across countries and cultural or socioeconomic groups, and multiperson (intergenerational) households have an increased transmission risk.^94^ Prevailing beliefs—for example, conspiracy or health-related beliefs—and values—for example, solidarity, responsibility—have been identified as crucial determinants of compliance with infection control measures during the COVID-19 pandemic.^41 95^ A similarly important determinant is the level of trust, especially in policy-makers and science.^96 97^ These aspects are closely related to health literacy, which can be impacted through policy measures in the longer run, but has to be taken as given in a medium time horizon. Health literacy has been shown to play an important role for individual risk behaviour.^98 99^ It is important to note that cultural factors do not only differ across countries but also within countries, as the pandemic has underscored existing inequalities and given rise to social divides.

Finally, international or exogenous factors have an increasing impact on health policy. The degree of international connectedness of individual countries or regions has significantly affected their epidemiological risk and thus the effectiveness of infection control measures.^100 101^

## Discussion

The COVID-19 pandemic put unprecedented pressure on health systems around the world, and its rapidly evolving nature for a long time primarily allowed decision-makers to react more than act. Several frameworks on health system resilience have been put forth, but in our view, most remain conceptual in nature and do not sufficiently acknowledge the role of different prerequisites and contextual factors each country faces. Prerequisites in our framework are traits of health systems that have been shown to improve resilience towards the shock of a pandemic. They also determine which and how well specific strategies can help manage a crisis. Contextual factors are less amenable to policy and will affect strategies like a filter.

The framework presented in this article was developed based on literature and in dialogue with stakeholders. Additionally, it was empirically enriched by using it in several case studies. Although these case studies only covered a rather limited selection of eight European countries, the practical application of the framework demonstrated its relevance and usefulness in the different country contexts.

The framework can be used as a tool for multiple purposes. Researchers can draw on the framework as a starting point to structure their analysis of a certain aspect of a health system that is embedded in specific contextual factors as well as stronger or weaker in its individual prerequisites. They may also use it to assess and compare resilience factors in different countries, as well as to investigate the transferability of strategies across countries.

Policy-makers can also make use of the framework: they can map their respective country in terms of contextual factors that will influence the effectiveness of policies and take stock of how far certain prerequisites for resilience have been established. This should help them identify barriers to implementation of certain policies or to analyse why a certain measure that was effective in a different country may not show the desirable effects in their own country.

What the framework in its current form cannot be used for is a quantification of health system resilience in the fashion of previously proposed scoring systems or indices.^5 6^ This would require operationalising the individual factors in numerical terms, which would be a challenging but undoubtedly interesting task.

While most contextual factors may not be amenable (or will take a long time) to change, some factors that are prerequisites for resilience can be improved during normal times to be better prepared for a future pandemic. To what extent this is the case will differ substantially between the individual factors as well as between health systems. For example, financing structures may be more rigid than some governance structures, and governance structures in one health system may be more difficult to reform than governance structures in a different health system. Contextual factors such as the legal system or political constellations once again play a major role in this regard. Thus, it is on policy-makers—with the help of the analytical framework presented in this article—to identify the policy areas where changes are possible and effective in the context of their health system. During a crisis, the analytical information provided by the framework can help assess the suitability, effectiveness and thus priority of specific policies.

Necessarily, our framework is limited to certain aspects that we found useful for analytical purposes. Other frameworks emphasise the cyclical nature of measures (similar, eg, to the ‘plan–do–check–act’ paradigm^102^), however, we see this as a given. It would have been an option to deviate more from the WHO health systems framework structure to more strongly emphasise certain aspects relevant to pandemics, but the WHO structure facilitates finding common ground between researchers and policy-makers. Several further aspects were considered to be added during the development process, but the more complicated a framework becomes, the less it is usable as a basis for analysis. Research into pandemic policies and politics will continue for quite some time and will bring further insights. This may require future adaptions of the framework and make some aspects more fine-grained. In-depth use cases for countries may also help tweak it and contribute to its evolution and usefulness.

All in all, our framework illustrates that there are numerous factors that need to be taken into account when designing policy during a pandemic. While some factors lie outside of the policy-maker’s influence at least in the medium term, many prerequisites have been shown to improve the resilience of a health system to exogenous shocks. These need to be addressed by policy-makers before the next pandemic hits. The existence of important contextual factors implies that there cannot be a one-size-fits-all approach, but knowledge of these factors may help adapt policies that were helpful in one country to the context of another.

## supplementary material

10.1136/bmjph-2023-000378 online supplemental file 1

## Data Availability

Data sharing not applicable as no datasets generated and/or analysed for this study.
