# Supplementary material for: What makes health systems resilient? An analytical framework drawing on European learnings from the COVID-19 pandemic based on a multitiered approach
Source: BMJ Public Health. 2024 Mar 14;2(1):e000378. doi: 10.1136/bmjph-2023-000378 (PMC11812772; doi:10.1136/bmjph-2023-000378)
Supplement: online supplemental file 1 [file bmjph-2-1-s001.pdf]

Summary table of main characteristics of the resilience frameworks identified in the literature review

	Focus	Geographic context	Phases	Structuring components
Chamberland-Rowe et al. (2019)	undefined health system instabilities	worldwide	1) pre-impetus<br>2) peri-impetus<br>3) post-impetus	• inputs<br>• mediators<br>• outputs
Gibson et al. (2012)	public health emergencies	USA	1) pre-incident<br>2) incident<br>3) post-incident	• prepare<br>• manage<br>• monitor<br>• investigate<br>• intervene<br>• recover
Haldane et al. (2021)	infectious diseases – COVID 19	worldwide	no explicit definition of phases	• governance and financing<br>• health workforce<br>• medical products and technologies<br>• public health functions<br>• health service delivery<br>• community engagement
Holloway et al. (2014)	infectious diseases – influenza pandemics	USA	1) investigation<br>2) recognition<br>3) initiation<br>4) acceleration<br>5) deceleration<br>6) preparation	• incident management<br>• surveillance and epidemiology<br>• laboratory<br>• community mitigation<br>• medical care and countermeasures<br>• vaccine<br>• risk communications<br>• state/local coordination
Khan et al. (2018)	public health emergencies	Canada	no explicit definition of phases	• governance and leadership<br>• planning process<br>• collaborative networks<br>• community engagement<br>• risk analysis<br>• surveillance and monitoring<br>• practice and experience<br>• resources<br>• workforce capacity<br>• communication<br>• learning and evaluation
McCabe et al. (2010)	public health emergencies	USA	no explicit definition of phases	• ready<br>• willing<br>• able

Stoto et al. (2017)	public health emergencies	Europe	no explicit definition of phases	• capacities<br>• response capabilities<br>• objectives
Thomas et al. (2020)	undefined shock	Europe	4-stage shock cycle:<br>1) preparedness<br>2) onset and alert<br>3) shock impact and management<br>4) recovery and learning	• governance<br>• financing<br>• resources<br>• service delivery
World Health Organization (2017)	health emergencies	worldwide	4-stage cycle:<br>1) assessing risks and capacity<br>2) planning<br>3) implementing<br>4) evaluating and correction	• governance<br>• capacities<br>• resources
World Health Organization (2019)	health emergencies and disasters	worldwide	no explicit definition of phases	• policies, strategies and legislation<br>• planning and coordination<br>• human resources<br>• financial resources<br>• information and knowledge management<br>• risk communications<br>• health infrastructure and logistics<br>• health and related services<br>• community capacities for health EDRM<br>• monitoring and evaluation

References

Chamberland-Rowe, C., Chiocchio, F., & Bourgeault, I. L. (2019). Harnessing instability as an opportunity for health system strengthening: A review of health system resilience. *Healthcare Management Forum*, *32*(3), 128–135.

Gibson, P. J., Theodore, F., & Jellison, J. B. (2012). The Common Ground Preparedness Framework: A Comprehensive Description of Public Health Emergency Preparedness. *American Journal of Public Health*, *102*(4), 633–642.

Haldane, V., De Foo, C., Abdalla, S. M., Jung, A.-S., Tan, M., Wu, S., Chua, A., Verma, M., Shrestha, P., Singh, S., Perez, T., Tan, S. M., Bartos, M., Mabuchi, S., Bonk, M., McNab, C., Werner, G. K., Panjabi, R., Nordström, A., & Legido-Quigley, H. (2021). Health systems resilience in managing the COVID-19 pandemic: Lessons from 28 countries. *Nature Medicine*, *27*(6), 964–980.

Holloway, R., Rasmussen, S. A., Zaza, S., & Cox, N. J. (2014). *Updated Preparedness and Response Framework for Influenza Pandemics* (MMWR Recommendations and Reports No. 63/1; Morbidity and Mortality Weekly Report, pp. 1–18). Centers for Disease Control and Prevention (CDC).

Khan, Y., O'Sullivan, T., Brown, A., Tracey, S., Gibson, J., Généreux, M., Henry, B., & Schwartz, B. (2018). Public health emergency preparedness: A framework to promote resilience. *BMC Public Health*, *18*(1), 1344.

McCabe, O. L., Barnett, D. J., Taylor, H. G., & Links, J. M. (2010). Ready, Willing, and Able: A Framework for Improving the Public Health Emergency Preparedness System. *Disaster Medicine and Public Health Preparedness*, *4*(2), 161–168.

Stoto, M. A., Nelson, C., Savoia, E., Ljungqvist, I., & Ciotti, M. (2017). A Public Health Preparedness Logic Model: Assessing Preparedness for Cross-border Threats in the European Region. *Health Security*, *15*(5), 473–482.

Thomas, S., Sagan, A., Larkin, J., Cylus, J., Figueras, J., & Karanikolos, M. (2020). *Strengthening health systems resilience—Key concepts and strategies* (Policy Brief No. 36; Health Systems and Policy Analysis). European Observatory on Health Systems and Policies.

World Health Organization. (2017). *A Strategic Framework for Emergency Preparedness*. World Health Organization.

World Health Organization. (2019). *Health Emergency and Disaster Risk Management Framework*. World Health Organization.
